# Optotracing for live selective fluorescence-based detection of *Candida albicans* biofilms

**DOI:** 10.3389/fcimb.2022.981454

**Published:** 2022-09-02

**Authors:** Elina Kärkkäinen, Saga G. Jakobsson, Ulrica Edlund, Agneta Richter-Dahlfors, Ferdinand X. Choong

**Affiliations:** ^1^ AIMES-Center for the Advancement of Integrated Medical and Engineering Sciences Karolinska Institutet and KTH Royal Institute of Technology, Stockholm, Sweden; ^2^ Department of Neuroscience, Karolinska Institutet, Stockholm, Sweden; ^3^ Fibre and Polymer Technology, KTH Royal Institute of Technology, Stockholm, Sweden

**Keywords:** optotracing, biofilm, Candida, cell wall, amyloid

## Abstract

*Candida albicans* is the most common fungal pathogen in humans, implicated in hospital-acquired infections, secondary infections in human immunodeficiency virus (HIV) patients, and is a significant contributor to the global antimicrobial resistance (AMR) burden. Early detection of this pathogen is needed to guide preventative strategies and the selection and development of therapeutic treatments. Fungal biofilms are a unique heterogeneous mix of cell types, extracellular carbohydrates and amyloid aggregates. Perhaps due to the dominance of carbohydrates in fungi, to date, few specific methods are available for the detection of fungal biofilms. Here we present a new optotracing-based method for the detection and analysis of yeast and biofilms based on *C. albicans* SC5314 as a model. Using commercial extracts of cell wall carbohydrates, we showed the capability of the optotracer EbbaBiolight 680 for detecting chitin and β-glucans. The sensitivity of this tracer to these carbohydrates in their native environment within fungal cells enabled the visualization of both yeast and hyphal forms of the microbe. Analysis of optotracer fluorescence by confocal laser scanning microscopy revealed extensive staining of fungi cell walls as well as the presence of intracellular amyloid aggregates within a subpopulation of cells within the biofilm. Further analysis of the photophysical properties of bound tracers by spectroscopy and spectral imaging revealed polymorphisms between amyloid aggregates within yeast and hyphal cells and enabled their differentiation. With exceptional spatial and temporal resolution, this assay adds a new technique that facilitates future understanding of fungal biofilms and their formation, and enables direct, unbiased diagnostics of these medically relevant biofilms, as well as the development of antifungal strategies.

## Introduction

*Candida albicans* (*C. albicans*) is among the most common fungal commensal and an opportunistic pathogen most frequently identified in fungal infections in humans by virtue of biofilm-formation (Mukaremera et al., 2017; Noble et al., 2017). Candidiasis, a fungal infection caused by *Candida spp*, is a substantial problem especially within immunocompromised individuals such as acquired immunodeficiency syndrome (AIDS) patients, who are susceptible to the opportunistic pathogens and readily acquire a systemic bloodstream infection induced by biofilm-formation (Merenstein et al., 2013; Noble et al., 2017). Indeed, 90% of AIDS patients develop oral candidiasis. Among *Candida spp*, *C. albicans* biofilms are a major contributor to the global antimicrobial resistance (AMR) burden with an increasing occurrence and a critically high mortality rate of 40% for systemic candidiasis (Noble et al., 2017; Pierce et al., 2017). Biofilms of *C. albicans* consist of sessile cells enclosed in a self-produced extracellular matrix (ECM) which provides stability and protects resident cells against external insults. This allows for the maintenance of homeostasis against incursions to the microenvironment that includes the host immune system and antifungal pharmaceuticals. As a result, fungal infections such as oral thrush, genital infections and systemic candidiasis are persistent and difficult to eradicate (Pierce et al., 2017).

In addition to the ability to form biofilms, *C. albicans* is known to possess exceptional morphological plasticity, being able to switch between various cell types with distinct morphologies that confer different functions in health and disease (Mukaremera et al., 2017). The unicellular growth state called yeast is the default in most *in vitro* conditions and involves white to opaque cells of shapes varying from perfectly round to ovals and ellipsoids. Yeast is thought to represent the dominant form of the commensal population in the microbiome. In contrast, the main cell type in the invasive *C. albicans* biofilms is multicellular hyphae, elongated tube-like cells that form branches and intertwine with each other (Noble et al., 2017). The cell wall composition of yeast and hyphae differs with hyphae having larger amounts of chitin and β-1,3-linked glucans, and less β-1,6-linked glucans. Differences in the secondary structure of these polysaccharides have also been reported between cell types. In particular, mannans in the hyphal cell wall exhibit a different morphotype compared to its counterpart in yeast cells that contributes to immune evasion (Lowman et al., 2003; Garcia-Rubio et al., 2019). While the initial growth by both lifestyles is accomplished by the adhesion of yeast to a surface, the subsequent propagation of the biofilm leading to infection occurs by switching to pseudohyphae, a transitional form between yeast and hyphae, as well as the intrinsically invasive hyphae. During the final stages of the biofilm yeast disperses from the structure and can migrate to new locations *via* the bloodstream causing disseminated disease (Pereira et al., 2021).

In addition to being a heterogeneous mix of different cell types, *C. albicans* biofilms are also known to contain a range of self-secreted ECM substances that contribute distinctly to the integrity and survivability of the overall culture. The ECM mainly consists of α-1,2-branched and α-1,6-mannans in mannan-glucan complexes associated with primarily β-1,6-glucans. The ECM also includes sparse β-1,3-glucans, as well as lipids and nucleic acids. Collectively, these compounds interact with each other to create a cohesive network, referred to as “fungal super glue” that provides mechanical stability, as well as adhesive interactions (Pierce et al., 2017). Furthermore, the ECM grants the enclosed cells increased resistance to antifungals mainly by preventing the drugs from reaching the cells by binding compounds of the matrix. Depending on the composition, the ECM can also confer the ability to evade and impair the host immune system by hampering access of immune cells to cell wall epitopes which are required for pathogen recognition and subsequent elimination (Pierce et al., 2017; Garcia-Rubio et al., 2019).

Despite the significance of the biofilm lifestyle in the fitness and virulence of *C. albicans*, few detection methods are available. Methods for analyzing *C. albicans* biofilms can be generally divided into measuring biomass such as with crystal violet, or metabolic activity as with XTT (2,3-Bis-(2-Methoxy-4-Nitro 5-Sulfophenyl)-2H-Tetrazolium-5-Carboxanilide) (Silva et al., 2008; Qu et al., 2020; Nett et al., 2011; Tsukatani et al., 2020). However, differentiating the growth states, which is crucial for identifying an active disease, has not yet been possible due to the obvious complexity of the *C. albicans* biofilm (Corte et al., 2019).

Optotracing utilizes a group of tracer molecules that emit no meaningful fluorescence in their unbound state. Binding to a target induces an on-like switching of the tracer alongside a unique fluorescence profile that can potentially identify each interaction (Choong et al., 2016a; Butina et al., 2020; Butina et al., 2022). With no reported toxicity against microbial and eukaryotic cells, optotracers enable real-time monitoring of culture growth and the transition from planktonic to a biofilm lifestyle (Choong et al., 2016a; Butina et al., 2022). Recently, we developed an optotracing method for the semi-high throughput detection and monitoring of *Salmonella* biofilms growth on air-solid interfaces (Choong et al., 2021). By comparing biofilms generated by wild type (wt) and isogenic biofilm mutants of *Salmonella enterica* serotype Enteritidis (*S. enteritidis*), we demonstrated the specificity of this method for biofilm detection, based on the tracing of ECM-curli (Choong et al., 2021). In parallel studies mapping carbohydrates in plant biomasses, we also reported the detection of cellulose and hemi-celluloses by optotracers (Choong et al., 2016b; Choong et al., 2019; Wahlström et al., 2020).

Herein we examine fungal biofilms for unique intracellular and extracellular organelles specific to this lifestyle that may function as a reporter, based on a *C. albicans* SC5314 model. Using the optotracer EbbaBiolight 680 (Ebba680), we also scrutinize the efficacy of optotracing for the detection of fungal biofilms.

## Materials and methods

### Strains, media, and supplements

*Candida albicans* wild-type strain SC5314 used in this study was maintained as stock at –80°C. Prior to experimentation, cultures were routinely prepared by streaking stock cultures on Sabouraud dextrose agar (SDA, Merck, Stockholm, Sweden) that were incubated at 37°C for 48 h after which they were kept in the fridge at 6°C. When required, overnight cultures of SC5314 were prepared by transferring 1 distinct colony from stock SDA plates into 5 mL of Sabouraud dextrose broth (SBD) and incubating the resulting suspension at 37°C for approximately 16 h. Purified fractions of chitin from shrimp shells (CAS no. 1398-61-4), a mixture of β-1,3-glucan and β-1,6-glucan marketed as ‘β-glucans’ (CAS no. 9012-72-0) and mannan (CAS no. 9036-88-8) from *Saccharomyces cerevisiae* were acquired from Sigma-Aldrich (Sweden). Ebba680, used as a media supplement for live tracing of yeast and biofilms was purchased from Ebba Biotech, Stockholm, Sweden.

### Yeast- and biofilm coverslip assays

To produce glass-adhered *Candida* yeast cells and biofilms, fresh 10^6^ CFU/mL suspensions of SC5314 were prepared by performing a 10 -fold dilution of overnight cultures in SDB and RPMI, respectively. 2 mL aliquots of each suspension were dispensed onto sterile coverslips (VWR, Stockholm, Sweden) in a 6-well plate (Merck, Stockholm, Sweden) and the setup was incubated at 37°C for 90 min for adhesion. After incubation, the coverslips were washed with phosphate buffer saline (PBS, Thermofischer Scientific, Stockholm, Sweden) and reincubated in 2 mL of sterile SDB and RPMI (Thermofischer Scientific, Stockholm, Sweden), respectively, at 37°C. After 72 h, yeast and biofilm colonized coverslips were washed with PBS and fixed with 2.5% glutaraldehyde (Merck, Stockholm, Sweden) for SEM. As indicated, the media was supplemented with EbbaBiolight 680 (Ebba680, 2 μL/mL) (Ebba Biotech, Stockholm, Sweden). Ebba680 is an oligothiophene-based structural probe that upon binding to macromolecules emits light in the red and far-red spectrum. The molecular structure of optotracers in the EbbaBiolight series is proprietary to the supplier.

### Scanning electron microscopy

The samples were fixed using 2.5% glutaraldehyde (Merck, Stockholm, Sweden) in 0.1 M phosphate buffer, pH 7.4. Fixed samples were adhered to a pore membrane and washed in MilliQ water prior to stepwise ethanol dehydration and critical-point-drying using carbon dioxide (Leica EM CPD300). The membranes were mounted on specimen stubs using carbon adhesive tabs and sputter coated with platinum (Quorum Q150T ES). SEM images were acquired using an Ultra 55 field emission scanning electron microscope (Zeiss, Oberkochen, Germany) at 3 kV and the SE2 detector.

### Fluorescence staining

For live chitin and β-glucans staining, a drop of calcofluor white stain (Merck, Stockholm, Sweden) containing Calcofluor White M2R (1 g/L) and Evans blue (0.5 g/L) was added directly to coverslip adhered fungal cells using a disposable Pasteur’s pipette and incubated for 5 min. Cell wall mannoproteins in coverslip adhered fungal cells were stained for 30 min with 50 ug/mL of Alexa488-conjugated Concanavalin A (ThermoFisher Scientific, Stockholm, Sweden) in 10 mM Na-HEPES (pH 7.4) with 2% D-glucose and washed with 10 mM Na-HEPES (pH 7.4) with 2% D-glucose. For vacuolar and membrane straining, coverslip adhered fungal cells were treated with 10 µM of MDY-64 (ThermoFisher Scientific, Stockholm, Sweden) in 10 mM Na-HEPES (pH 7.4) with 2% D-glucose for 4 min and washed with 10 mM Na-HEPES (pH 7.4) with 2% D-glucose. Amyloid proteins were stained with 40 µg/ml of Thioflavin S in PBS for 8 min, and washed with PBS. Nuclei of cells were stained with 2.5 µg/mL 4′,6-diamidino-2-phenylindole (DAPI) in 10 mM Na-HEPES (pH 7.4) with 2% D-glucose for 15 min and washed with 10 mM Na-HEPES (pH 7.4) with 2% D-glucose. All coverslips were mounted with DAKO and sealed on a glass slide with nail polish before microscopy.

### Airyscan fluorescence microscopy

Confocal fluorescence microscopy was performed at Biomedicum imaging core facility (Karolinska Institutet, Sweden). The imaging of *C. albicans* was done with Zeiss LSM900 Airy using 63x oil objective and the Airyscan detector. Fluorescence for Calcofluor White, DAPI, Alexa488-conjugated Concanavalin A was collected using the corresponding preset settings of filters in the software. Signals for MDY-64 and Thioflavin S were collected using preset settings of filters for EGFP and Alexa488 respectively. To detect Ebbabiolight 680 fluorescence, software preset settings of filters for propidium iodide were applied.

### Preparation and analysis of images

Fiji (ImageJ2 Version: 2.3.0/1.53q, Wisconsin, USA) was used to process images acquired after airyscan-processing to display optimal signal intensity for clear images with minimum visible noise. This was done by adjusting the minimum and maximum found under Image>Adjust>Brightness/contrast. Further post-imaging analysis of images to reveal weak Ebba680 fluorescence was done by adjusting the minimum and maximum in the same function to display relatively weaker signals located at the cell walls.

### Optotracer-based detection of chitin, glucan, and mannan by fluorescence spectroscopy

To prepare polysaccharide samples for fluorescence spectroscopy, a stock solution of 2 mg/mL for each compound was prepared in PBS. Additionally, a 2 µL/mL stock solution of Ebba680 was prepared in PBS. Then, two-fold serial dilutions were performed as triplicates in a 96-well plate using PBS with 100 µL end-volume per well. After this, 100 µL of Ebba680 stock solution was added to designated wells to a final concentration of 1 µL/mL, otherwise, PBS was added. Fluorescence spectroscopy of polysaccharides with and without Ebba680 was performed using SynergyMx plate reader (Biotek, Sweden). The following settings were used: excitation at 350-600 nm, emission collected at 650 nm; emission at 550-850 nm using 500 nm excitation, at 1 nm steps with 102% sensitivity. Signals collected were analyzed and presented using GraphPad.

### Optotracer-based detection of *C. albicans* by fluorescence spectroscopy

To produce yeast- and biofilm samples for end-point fluorescence spectroscopy in a 96-well microtiter plate format, overnight cultures of SC5314 were diluted 10-fold to achieve a culture density of 10^6^ CFU/mL, with SDB and RPMI respectively. As indicated, the media was supplemented with Ebba680, 1 μL/mL (Ebba Biotech, Stockholm, Sweden). 100 µL of each suspension was inoculated in triplicate into a transparent flat bottom 96-well microtiter plate. Both yeast- and biofilm cultures were incubated at 37°C for 19 h, after which fluorescence signals were recorded using a SynergyMx plate reader (Biotek, Sweden). The following settings were used: excitation at 350-600 nm, emission collected at 650 nm; emission at 550-850 nm using 500 nm excitation, at 1 nm steps with 102% sensitivity. Signals collected were analyzed and presented using GraphPad.

### Spectral imaging analysis

Spectral analysis was performed on Ebba680 traced yeast cells and biofilms adhered on glass coverslips using an inverted Zeiss LSM710 confocal microscope and a 63x oil objective. Signals from amyloid aggregates bound Ebba680 when excited at 458 nm were collected with an emission range of 420-721 nm in 9.7 nm steps. Spectra from 6 to 19 regions of interest per sample were selected and analyzed using GraphPad (**Supplementary Figure 8**).

### Statistical analysis

Data were organized and processed with GraphPad Prism 9 Version 9.4.0 (453) (Graphpad software, La Jolla, CA, USA) (Swift, 1997).

## Results

### Development and validation of a method for *C. albicans* biofilm growth

**Figure 1:**
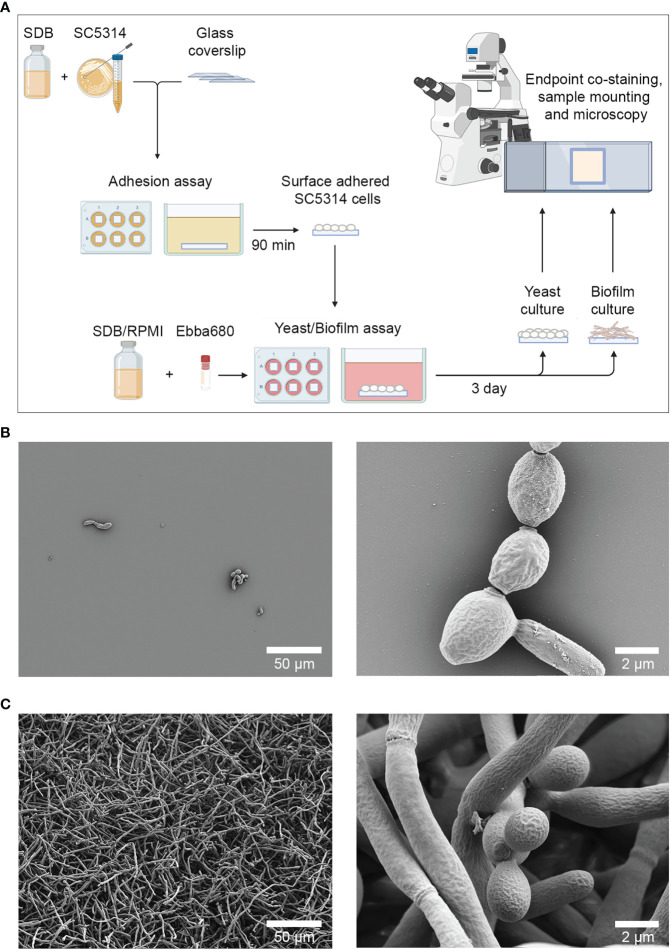
Cultivation of surface adhered *C. albicans* yeast cells and biofilms. **(A)** Workflow for producing yeast cells and biofilms. Sterile glass coverslips were immersed in 2 mL of *C. albicans* in SDB for 90 min at 37°C, after which coverslips containing adhered cells were re-incubated in 2 mL of SDB or RPMI with or without Ebba680 in 6 well plates for 3 days at 37°C. Surface adhered yeast cells and biofilms were subsequently analyzed by scanning electron microscopy, immunofluorescence techniques, confocal microscopy and spectral imaging analysis. **(B)** Scanning electron micrographs of surface adhered yeast cells at low (left) and higher magnifications (right). **(C)** Scanning electron micrographs of biofilm cells at low (left) and higher magnifications (right). Representative images of three independent experiments are shown.

To develop a biofilm-specific detection method based on optotracing, a *C. albicans* biofilm model was set up based on the adhesion assay described by Jin et al., 2004, and the biofilm induction method described in Weerasekera et al., 2016 (Jin et al., 2004 *;* Weerasekera et al., 2016). Sterile glass coverslips were immersed in 2 mL suspensions of *C. albicans* SC5314 grown in SDB at 37°C to allow cells to adhere. After 90 min, coverslips were washed, and re-incubated in 2 mL of SDB and/or RPMI at 37°C for an additional 72 h to allow the development of yeast cells and biofilms respectively (**Figure 1A**). To verify the development of these morphologies, samples were fixed in 2.5% glutaraldehyde and visualized by SEM. *C. albicans* grown in SDB were found sparely adhered to the glass coverslips (**Figure 1B**). Cells were mostly ovoid, with a small number of elongated cells that may be in the process of division. While ECM substances were occasionally seen on the surface of yeast cells, no large hyphal growths were observed. Collectively, this implied dominance of yeast cells and a lack of biofilm formation. In contrast, *C. albicans* grown in RPMI formed an extensive network of hyphal cells and was a clear indication of biofilm formation (**Figure 1C**). A closer examination showed a small population of ovoid-shaped yeast cells that may be part of the budding process. No ECM was visible in this sample. The distinct morphological differences between *C. albicans* growth in SBD and RPMI confirmed the validity of this method for generating single cell and biofilm samples for comparison in this study. From this point on, *C. albicans* cultures developed from growth in SDB are referred to as yeast cells, while cultures grown in RPMI are referred to as biofilms.

### Comparative immunofluorescence analysis of yeast cells and biofilms by standard biofilm detection methodologies

**Figure 2:**
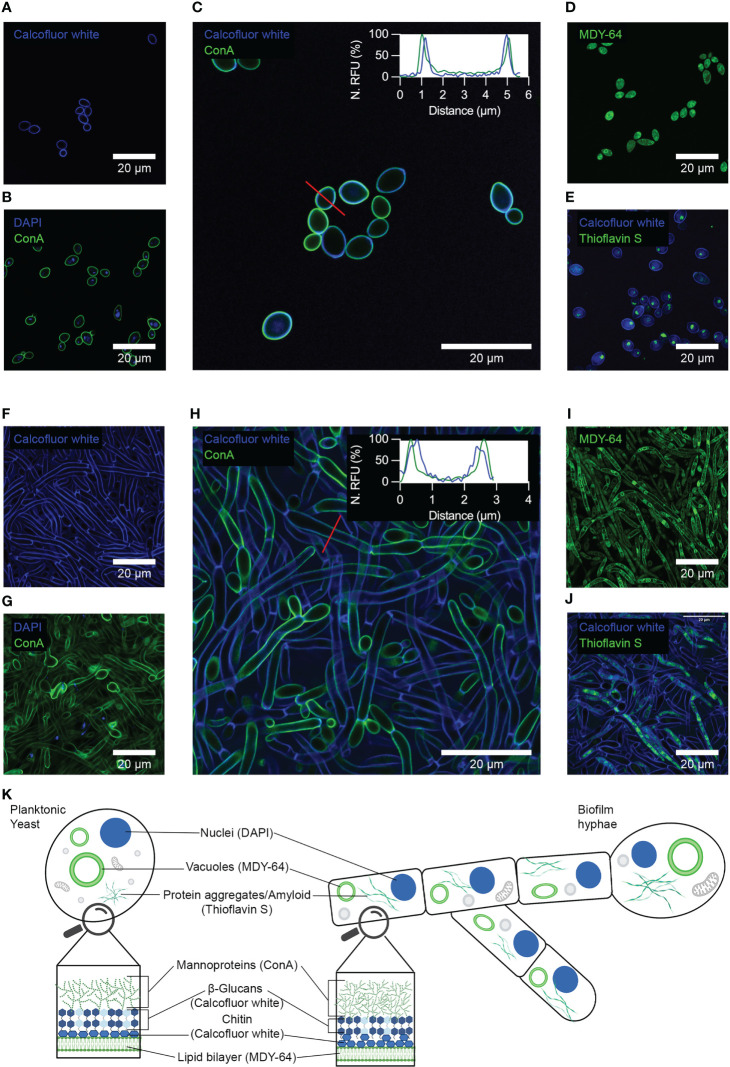
Immunofluorescence analysis of yeast cells and biofilms using conventional molecular probes for fungi. Confocal microscopy showing yeast cells stained with **(A)** calcofluor white, **(B)** DAPI and Alexa488 conjugated Concanavalin A (ConA), **(C)** calcofluor white and ConA, **(D)** MDY-64, and **(E)** calcofluor white and thioflavin S. Confocal microscopy of biofilms stained with **(F)** calcofluor white, **(G)** DAPI and ConA, **(H)** calcofluor white and ConA, **(I)** MDY-64, and **(J)** calcofluor white and thioflavin S. The embedded graphs in **C** and **H** show normalized fluorescence intensities of calcofluor white and ConA plot as a line profile. Red lines show the location of the line profile. **(K)** Schematic summary of the immunofluorescence analyses of yeast cells and biofilms, showing materials and organelles detected and the corresponding molecular probe used. Representative images of three independent experiments are shown.

To identify components of *C. albicans* biofilm that can function as specific indicators unique from the single cellular state, we performed a detailed immunofluorescence analysis of yeast cells and biofilms using a range of probes for cell wall polysaccharides, glycoproteins, nucleic acids, lipids and proteins. The staining of yeast cells with calcofluor white, a compound that binds to chitin and β-glucans showed homogenous staining of materials in the cell wall (**Figure 2A**). Application of the DNA stain DAPI showed the presence of spherical-shaped organelles, characteristic of nuclei, intracellularly located as indicated by co-staining with Alexa488 conjugated Concanavalin A (ConA) (**Figure 2B**, **Supplementary Figures 1A–C**). Co-staining with ConA and calcofluor white showed the localization of these stains to the cell wall, which partially overlapped (**Figure 2C**, **Supplementary Figures 2A–C**). Analysis of the fluorescence intensities of calcofluor white and ConA on a line profile showed that ConA signals were externally located in contrast to calcofluor white. This pattern of staining was consistent with the previously described organization of mannoproteins, β-glucans and chitin in the cell wall (Garcia-Rubio et al., 2019). Staining with the membrane marker MDY-64 showed the presence of both the plasma membrane, as well as tonoplasts within the cells (**Figure 2D**). Finally, staining with thioflavin S showed both densely packed and dispersed amyloids (**Figure 2E**, **Supplementary Figures 3A–C**). Co-staining of the cell wall with calcofluor white indicated that these organelles were intracellular.

Immunofluorescence analysis of biofilms with calcofluor white showed localization of the stain to the cell walls of hyphal cells (**Figure 2F**). Septa formed within these elongated cells were also visible. Co-staining of this biofilm with DAPI and ConA showed the presence of intracellular spherical-shaped nuclei (**Figure 2G**, **Supplementary Figures 1D–F**). Not all cells within the field of view presented a nucleus, likely due to the great length of these filaments. Subsequent co-staining of biofilm cells with ConA and calcofluor white showed partial overlap of their signals and localization of these stains to the cell wall (**Figure 2H**, **Supplementary Figures 2D–F**). Analysis of the fluorescence intensities by plotting the line profile showed that ConA signals were externally located in contrast to calcofluor white. Staining with the membrane marker MDY-64 showed the presence of both the plasma membrane as well as tonoplasts within both ovoid cells and hyphae (**Figure 2I**). Co-staining with thioflavin S and calcofluor white showed the presence of both densely packed and dispersed organelles throughout the intracellular compartment of all cell morphologies (**Figure 2J**, **Supplementary Figures 3D–F**).

In summation, immunofluorescence analysis revealed no clear cellular or extracellular structure that could function as specific indicators of the biofilm state (**Figure 2K**). However, tracing the difference in the relative abundance of cell walls and materials between yeast and biofilm morphology may be a possible strategy for differentiating the biofilm from yeast cells.

### Ebba680 binds and detects purified fungi cell wall polysaccharides

**Figure 3:**
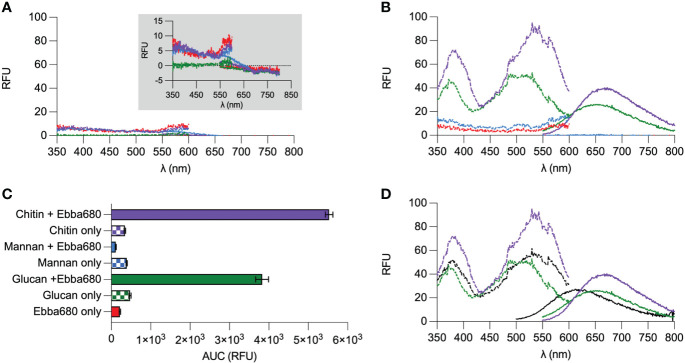
Optotracing of pure commercially available cell wall components. **(A)** Spec-plot of Ebba680 in free form (red), chitin (purple), mannan (blue) and β-glucans (green). A zoom-in of the spectra by reducing the range of the Y-axis is shown in the inner panel. **(B)** Spec-plot of Ebba680 in free form (red, as first shown in **(A)** and Ebba680 when mixed with chitin (purple), mannan (blue) and β-glucans (green). **(C)** The total fluorescence intensity, calculated as the area under the curve in the emission spectra (AUC) of Ebba680 in mixed with chitin (purple), mannan (blue), β-glucans (green), as well as the free form (red), is shown in a bar chart. **(D)** Spec-plot of Ebba680 when mixed with a heterogenous mixture with equal amounts of mannan, chitin, and β-glucans. Spectra of Ebba680 mixed with chitin and β-glucans (green) and as first shown in **(B)** is included for comparison. All lines and bars show mean of n=3, with three technical repeats. In **(C)**, the standard deviation is shown. Dashed lines in spec-plots indicate the excitation spectra (dash), while solid lines indicate the emission spectra.

As common markers failed to differentiate yeast cells from biofilms, we tested if optotracers could function as an alternative. To predict if optotracers could function as tracers of fungi, we first screened Ebba680 against commercially available purified cell wall polysaccharides. Specifically, chitin from shrimps, mannans from *S. cerevisiae*, and a mixture of β-1,3-glucan and β-1,6-glucan (marketed ad herein referred to as β-glucans) from *S. cerevisiae* were obtained. Equal volumes of Ebba680 (2 µL/mL) were mixed with suspensions of each polysaccharide, after which the photophysical properties were recorded in a plate reader and visualized spectral plot showing the relative fluorescence unit (RFU) recorded at each wavelength of excitation and emission. In the absence of Ebba680, neither mannan, β-glucans nor chitin emitted any notable amount of fluorescence across all wavelengths (**Figure 3A**). In the absence of a binding target, the free form of Ebba680 absorbs and emits weakly across a wide range of wavelengths. Further examination by replotting this data in a normalized spectral plot (N. Spec. plot), in which we assigned 0% to the lowest and 100% to the highest recorded RFU, showed no clear wavelengths of maximum absorption (Ex. λmax) albeit relatively higher RFUs emitted when the tracer is excited in the ranges of > 385 nm and < 560 nm (**Supplementary Figure 4A**). Similarly, no clear wavelength of maximum emission (Em. λmax) was observed for the free form of Ebba680. A simple summation of all fluorescence detected in the emission spectrum by calculating the area under the curve (AUC) revealed a value of 218 RFU ± 16, which represents the native fluorescence of Ebba680’s free form and the tracer’s ‘off’ state (**Figure 3C**). When combined with mannan, a minor increase in RFU was detected across all wavelengths of excitation and emission (**Figure 3B**). Comparing this spectral pattern with Ebba680 in its unbound state on a N. Spec-plot revealed a high degree of similarity, suggesting that Ebba680 did not detect mannan (**Supplementary Figure 4B**). The AUC of 111 RFU ± 14 emitted by Ebba680 in the presence of mannan was lower than unbound Ebba680 and mannan alone (390 RFU ± 16, **Figure 3C**). When mixed with β-glucans, Ebba680 exhibited a Ex. λmax at 495 nm ± 32, with a secondary peak at 373 nm ± 11 and was distinctly different from unbound Ebba680 and β-glucan alone. The AUC of β-glucans bound Ebba680 was 3828 RFU ± 164, and approximately 17.5 -fold higher than unbound Ebba680 (**Supplementary Figure 4C**). The appearance of a unique spectral profile and on-like switching of fluorescence is indicative of the binding between Ebba680 and β-glucans, as well as an associated change in the tracer’s molecular backbone. When incubated with chitin, Ebba680 showed an Ex. λmax of 537 nm ± 28, with a secondary peak at 382.5 nm ± 7. This spectral signature was distinct from that of chitin alone, unbound Ebba680, as well as β-glucans bound Ebba680. The AUC of chitin-bound Ebba680 was 5529 RFU ± 94, and approximately 25.4 -fold higher than that of the tracer alone. This on-like switching of fluorescence together with the spectral profile is indicative of binding between Ebba680 and chitin, originating from a change in the tracer’s molecular backbone during this interaction. Since all 3 polysaccharides are present in the cell wall of fungi, we added Ebba680 to a sample composed of equal amounts of mannan, β-glucans and chitin and recorded the photophysical quality of the tracer. In this instance, Ebba680 exhibited an Ex. λmax and Em. λmax to 543.5 nm ± 30, accompanied by a secondary peak at 376 nm ± 15 (**Figure 3D**). Comparing this spectral profile revealed a great degree of similarity to that of chitin-bound Ebba680, implying that chitin had the highest affinity for the tracer compared to the mannan and β-glucans. Collectively, the aforementioned analyses of the photophysical properties of Ebba680 in the presence of purified cell wall polysaccharides revealed binding to β-glucans and chitin, two of the three known components, hence a strong indication that this tracer molecule will be effective in detecting fungi.

### Optotracing by Ebba680 allows visualization of yeast cells and biofilms

**Figure 4:**
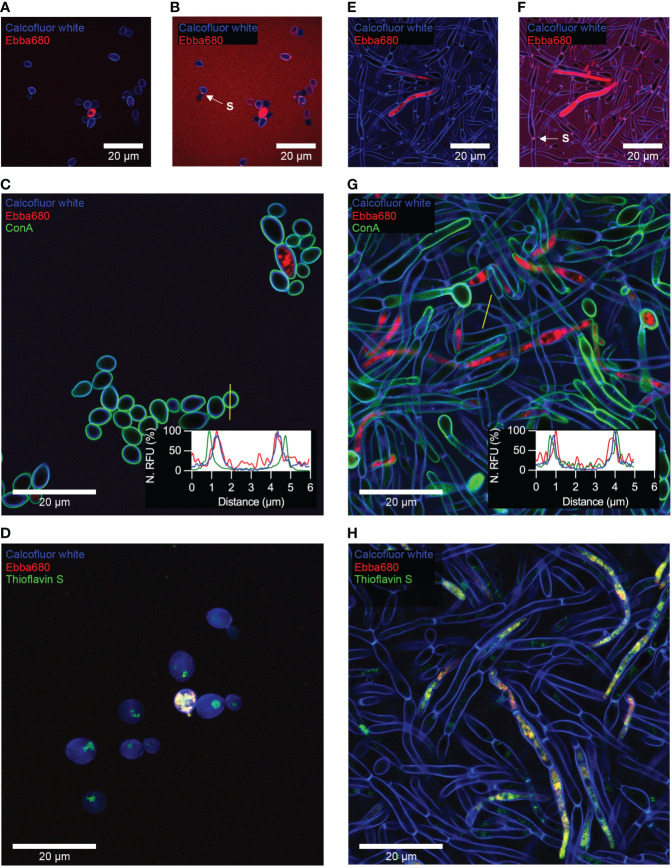
Optotracing of yeast cells and biofilms using Ebba680. Confocal microscopy of surface attached yeast cells grown from 72h incubation in Ebba680 supplemented SDB, co-stained with **(A, B)** calcofluor white, **(C)** calcofluor white and ConA and **(D)** calcofluor white and Thioflavin S. **(B)** Post-imaging enhancement of Figure **A** to reveal Ebba680 bound cell walls. Confocal microscopy of surface attached biofilm grown from 72 h incubation in Ebba680 supplemented RPMI, co-stained with **(E, F)** calcofluor white, **(G)** calcofluor white and ConA and **(H)** calcofluor white and Thioflavin S. **(F)** Post-imaging enhancement of Figure **E** to reveal Ebba680 bound cell walls in the biofilm. Septae, dividing connected cells in Figures **B** and **F** are indicated by an arrow and denoted ‘S’. The embedded graphs in **C** and **G** show normalized fluorescence intensities of Ebba680, calcofluor white and ConA plot as a line profile. Yellow lines show the location of the line profile. Representative images of three independent experiments are shown.

To determine if Ebba680 could indeed detect cell polysaccharides in their native state, we applied this tracer to stain yeast cells and biofilms. *C. albicans* adhered glass coverslips produced from the adhesion assay, were incubated in Ebba680 supplemented SDB and RPMI to allow the tracers to integrate into cells as they propagate. After incubation, coverslips were washed with PBS and stained with calcofluor white to delineate the fungal cell walls. Recording of Ebba680 signals, using standard settings for propidium iodide (PI), alongside calcofluor white from traced yeast cells showed distinct red fluorescence localized to intracellular bodies of a portion of cells within the sample (**Figure 4A**, **Supplementary Figures 5A–C**). Fluorescence within the cell wall was only visible upon further post-imaging analysis of Ebba680 signals by reducing the dynamic range of acquired micrographs in ImageJ. In enhanced fluorescence micrographs, Ebba680 fluorescence overlapped with calcofluor white (**Figure 4B**, **Supplementary Figures 5D, E**). To further evaluate the sensitivity of Ebba680 for cell wall components, yeast cells traced with Ebba680 were stained with both calcofluor white and ConA (**Figure 4C**). Analysis of the location of each stain by plotting signals recorded in a line profile revealed clear co-localization of calcofluor white and Ebba680 fluorescence. This result also indicates that Ebba680 was capable of binding and detecting chitin and β-glucan in its native environment. No co-localization was observed between ConA and Ebba680, confirming the lack of sensitivity of the tracer for mannoproteins. Recalling earlier findings of the immunofluorescence analysis shown in **Figure 2** hinted at similarities between intracellular bodies traced by Ebba680 and amyloid fibers detected by thioflavin S. As such, yeast cells grown in Ebba680 supplemented SDB were co-stained with thioflavin S. Calcofluor white was also used to outline the cell wall as a reference. Recording of these signals under standard settings for Calcofluor white, Alexa488 and PI revealed a co-localization of Ebba680 fluorescence with a subset of thioflavin S stained intracellular bodies, confirming that these were amyloid proteins (**Figure 4D**, **Supplementary Figures 6A–D**).

Biofilms stained with Ebba680 and calcofluor white illuminated intracellular objects with distinct red fluorescence in a small number of hyphal cells when imaged (**Figure 4E**, **Supplementary Figures 5F–H**). These were large with both dense and sparsely morphologies. Ebba680 fluorescence localized to the cell wall, coinciding with calcofluor, was only visible during the post-imaging analysis of Ebba680 signals upon reducing the dynamic range of acquired micrographs in ImageJ (**Figure 4F**, **Supplementary Figure 5I and J**). Further analysis of the sensitivity of Ebba680 for cell wall components, by plotting signals recorded from a sample co-stained with calcofluor white and ConA in a line profile, showed poor co-localization with ConA. In the case of calcofluor white and Ebba680, signals partially overlapped, implying that while the two stains shared common targets, likely chitin and β-glucan, Ebba680 may stain additional components located towards the inner face of hyphal cell walls (**Figure 4G**). Finally, co-staining of biofilms with thioflavin S showed the co-localization of Ebba680 fluorescence with a subset of thioflavin S stained amyloid proteins (**Figure 4H**, **Supplementary Figures 6E–H**).

Collectively, our model shows that Ebba680 can detect yeast cells and biofilms through the tracing of cell walls and intracellular amyloid proteins. However relatively weak signals from the cell wall are likely to be easily missed in favor of more productive binding targets such as amyloid proteins in this instance. As amyloid proteins were not present in all cells, detection of *C. albicans via* intracellular amyloid proteins may not result in complete detection of all cells present. On a positive note, partial colocalization of Ebba680 to more diffused organelles in hyphal cells suggests that this tracer may be capable of differentiating intracellular types of amyloid aggregates. Comparison of cell wall located Ebba680 signals between yeast and hyphal cells also revealed possible differences in cell wall composition between the cell types.

### Spectrophotometric and spectral imaging analysis by Ebba680 allows differentiation of yeast cells and biofilms

While optotracing of yeast and hyphal cells detected visible differences that could be used to identify biofilms by microscopy, we hypothesized that spectrophotometry of optotracers may be a better alternative due to the quantitative data it produces. For this purpose, overnight cultures of *C. albicans* in SDB were diluted 10-fold and re-inoculated into SDB and RPMI containing 1 µL/mL of Ebba680 and introduced into a sterile flat bottom 96 well plate format in 100 µL aliquots. Plates were incubated at 37°C for 19 h to generate yeast cells and biofilms, after which the photophysical properties of Ebba680 were measured and visualized in a N. spectral plot. In parallel, yeast cells and biofilms were grown without Ebba680 for comparison.

**Figure 5:**
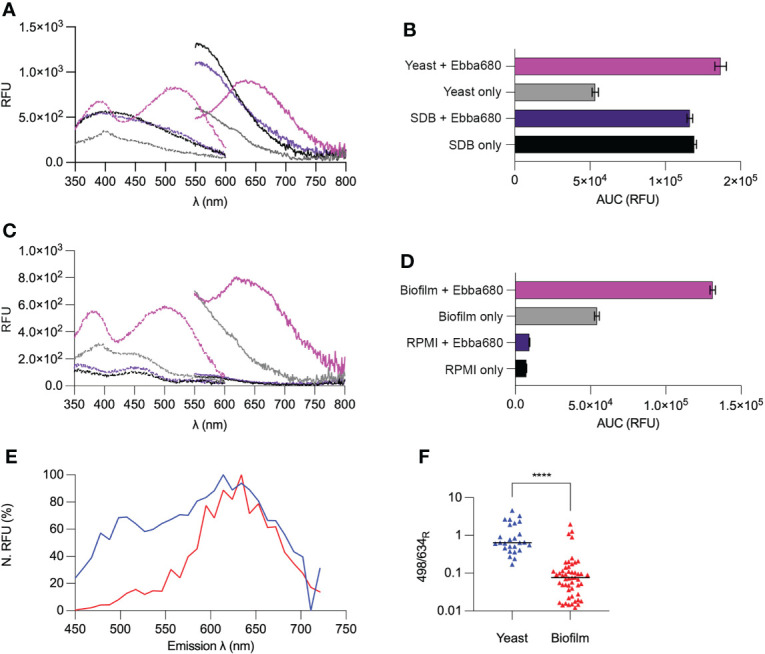
Optotracing yeast cells and biofilms. **(A)** Spec-plot of SDB (black), Ebba680 in SDB (purple), yeast cells (gray) and yeast cells in Ebba680 supplemented SDB (pink). **(B)** The total fluorescence intensity, calculated as the area under the curve in the emission spectra (AUC) of SDB (black), Ebba680 in SDB (purple), yeast cells (gray) and yeast cells in Ebba680 supplemented SDB (pink). **(C** Spec-plot of RPMI (black), Ebba680 in RPMI (purple), biofilms (gray) and biofilms in Ebba680 supplemented RPMI (pink). **(D)** The total fluorescence intensity, calculated as the area under the curve in the emission spectra (AUC) of RPMI (black), Ebba680 in RPMI (purple), biofilms (gray) and biofilms in Ebba680 supplemented RPMI (pink). All lines and bars show mean of n=3, with 3 technical repeats. In **(C, D)**, the standard deviation is shown. Dashed lines in spec-plots indicate the excitation spectra (dash), while solid lines indicate the emission spectra. **(E)** Spectral imaging analysis of intracellular amyloid bodies showing the emission spectra of regions of interest from intracellular amyloid aggregates from yeast (blue) and biofilms (red), shown in a Normalized Spec-plot. **(F)** Plot of the ratio of emission intensities at wavelengths of 498 and 634 nm for Ebba680 following binding to intracellular amyloid aggregates in yeast (blue triangles) and biofilm (red triangles). The mean of all ROIs is shown. Data from three three independent experiments are shown. **** denotes a P-value of < 0.0001.

In the absence of *C. albicans*, the spectral properties of SDB, and Ebba680 supplemented SDB were found to be highly similar, presenting an Ex. λmax of 407.5 nm ± 24 and an Ex. λmax of 391 nm ± 15 respectively (**Figure 5A**). The AUC of SDB was 119151 RFU ± 1524, indicative of a significant degree of autofluorescence within this growth medium. SDB containing Ebba680 exhibited a similar AUC of 116294 RFU ± 1928, implying that the tracer was in its off-state (**Figure 5B**). Yeast cells absorbed and emitted across a wide range of wavelengths, showing an Ex. λmax of 399.5 nm ± 6. The AUC of 53458 RFU ± 2128 detected, circa 0.45 fold lower than that of SDB and Ebba680 supplemented SDB, suggests a possible depletion of auto-fluorescent components within SDB during the culture’s growth (**Supplementary Figure 7A**). Analysis of Ebba680 traced yeast cells showed the combined presence of all fluorescence previously detected in yeast cells and SDB. In addition to the Ex. λmax located at 514 nm ± 6, a secondary peak at 388 nm ± 19 was observed. The accompanying increase in AUC to 136747 RFU ± 3817, 1.18 -fold higher than that of Ebba680 supplemented SDB implied an on-like switching of the tracer, indicative of the binding and detection of yeast cells.

RPMI possessed distinct autofluorescence across a wide range of wavelengths, showing a high degree of absorbance < 360 nm, an Ex. λmax of 458 nm ± 7, and an AUC of 7330 RFU ± 24 (**Figures 5C, D**). RPMI supplemented with Ebba680 exhibited an Ex. λmax of 447 nm ± 7 and a spectral pattern identical to that of RPMI. The AUC of 9376 RFU ± 253, which was similar to that of RPMI indicated that the tracer was in its off-state. Biofilms grown in RPMI were found to have a distinct degree of autofluorescence across a wide range of wavelengths, showing an Ex. λmax of 404 nm ± 22 nm, and an AUC of 54261 RFU ± 1622. Comparing these spectra with that of cultures in SDB implied that yeast cells within the biofilm were the likely major source of autofluorescence. Ebba680 traced biofilms absorbed and emitted across a wide range of wavelengths, showing an Ex. λmax of 508 nm ± 19 and a second region of high absorbance at 392 nm ± 5. The AUC of these Ebba680 traced biofilms was 131103 RFU ± 1767, 2.4 -fold higher than biofilms alone, indicating an on-like switching of the tracer (**Supplementary Figure 7B**). In summation, the above spectrophotometric analysis of Ebba680 fluorescence revealed distinctly different spectral profiles when bound to yeast cells and biofilms, thereby allowing their differentiation.

To determine if the differentiation of biofilms from yeast cells by optotracing was due to differences in intracellular amyloid proteins present within cell types of each lifestyle, spectral imaging analysis was performed to identify differences in the photophysical properties of bound tracer molecules (**Figure 5E**, **Supplementary Figures 8A, B**). Signals collected from a total of 26 regions of interest (ROI) in yeast cells and 51 ROI in hyphae from three independent experiments, representing Ebba680 stained amyloid aggregates in yeast and hyphae were analyzed in a N. Spec-plot. In yeast cells, amyloid aggregate bound Ebba680 exhibited prominent fluorescence across a wide range of wavelengths, with an Em. λmax of circa 614 nm. In contrast, Ebba680 bound to amyloid aggregates in biofilm hyphal cells emitted at distinctly longer wavelengths, presenting an Em. λmax of circa 634 nm. On average, Ebba680 bound to amyloid aggregates in biofilm hyphal cells presented lower N. RFU values below 600 nm. To assess the results in more detail, and to visualize the spectral distribution of each sample, the ratios of fluorescence intensity at 498 nm and 634 nm (498/634_R_) were calculated (**Figure 5F**). Ebba680 bound amyloid proteins in yeast showed significantly higher mean 498/634_R_ values than biofilm samples, indicating an emission range at shorter wavelengths compared to Ebba680 bound amyloid proteins in biofilms. Collectively, these dissimilarities in emission patterns of Ebba680 reflect differences in the tracer’s photophysical state, suggesting the polymorphism of intracellular amyloid aggregates between yeast cells and biofilms.

## Discussion

In this study, we showed the development of an optotracer-based method for the detection and differentiation of *C. albicans* yeast cells and biofilms using the clinical strain SC5314 as a model. The process is relatively simple, requiring only small sample volumes and fairly common spectrophotometers. Central to this method is the use of the fluorescence tracer molecule, Ebba680, supplemented into the growth medium SDB and RPMI, that transitions from an off- to an on-like state when a target appears. Two likely targets were revealed in this study, intracellular amyloid proteins, as well as the cell wall. Furthermore, we were able to attribute the differentiation of yeast cells and biofilms to the polymorphism of intracellular amyloid proteins across cell types associated with each lifestyle with spectral imaging analysis. This was apparent in both visual and non-visual methods, and the first demonstration of the optotracing of fungal cells.

As with a new methodology, we observed several limitations to this application of optotracing. Ebba680 fluorescence from cell walls was substantially weaker than signals from intracellular amyloid proteins and was not visible prior to post imaging enhancements. Such weak signals could easily be missed in the presence of more prominent fluorescence both when multiple targets are present for a tracer molecule, as well as when multiple stains are applied. Since no biofilm-specific organelles were detected during fluorescence imaging, the differentiation of yeast cells and biofilms was dependent on profiling the tracer molecule’s photophysical properties *via* its absorption and emission properties. Optotracing of *Candida* cultures thus inherits the limitations of spectrophotometry, as well as spectral imaging analysis. Spectrophotometry records all fluorescence within a sample and only within the instruments’ ‘field of view’. It is therefore important to include a robust panel of references to identify background signals. Adequate positioning of samples is also essential and can be extremely challenging in live studies of biological systems. On the other hand, spectral imaging can be subjective as the technique is dependent on visual analysis and identifying ROIs. Without rigorous experimentation, results may not be representative of the entire sample. In addition, optotracing and thioflavin S staining showed that the presence of intracellular amyloid proteins was not ubiquitous across all cells in both yeast and biofilm samples. As the differentiation of these lifestyles by optotracing was attributed to the detection of different types of intracellular amyloid proteins, further examination to their frequency and identity is required.

To date, few tools are available for the detection of fungal biofilms. This may be due to the prominent presence of carbohydrates that are conventionally difficult to detect. Furthermore, in contrast to bacterial biofilms that express ECMs with distinct biological molecules such as amyloid curli proteins and DNA, biofilms of *C. albicans* are an extensive weave of yeast, hyphal and pseudohyphal cells, with a small amount of mannoproteins and glucans (Bain et al., 2014; Machová et al., 2015). Detection of *C. albicans* biofilm must therefore be based on detecting the very intricate differences in the presence and quantity of these cell types (Gulati and Nobile, 2016; Noble et al., 2017). Optotracing-based analysis of fungi samples, a method capable of automated high-throughput detection and differentiation of cell types may reduce the occurrence of user biases and become a useful addition to the toolbox of fungal research.

In both bacterial and fungal biofilms, extracellular amyloid protein aggregates are integral in the formation of biofilms. In this study, the amyloid protein aggregates identified by both thioflavin S and Ebba680 staining were localized exclusively to the intracellular compartment. In *Candida*, the Als family of adhesins are among the most well-understood and play an integral role in fungal biofilm formation. These are a group of surface-located functional structures organized into hundreds of clusters that mediate adhesion to a diverse range of biotic and abiotic surfaces (Bouyx et al., 2021). The surprising absence of surface-located Als amyloid proteins and others may be specific to our model and requires further examination of why these were not expressed. However, how amyloid proteins are used by *C. albicans* to develop biofilms is still poorly understood at the molecular level and may benefit from tools such as those developed in this study. Our analysis of *C. albicans* biofilms revealed differences in Ebba680 traced amyloids that were detectable by spectroscopy and spectral image analysis, suggesting the simultaneous presence of different types of amyloid proteins. As methods to analyze and differentiate types of amyloid proteins have been developed in the study of disease-associated amyloid aggregates, the findings of this study open new avenues in research (Nilsson et al., 2007; Ostapchenko et al., 2010; Fändrich et al., 2018; Klingstedt et al., 2019). This includes the determination of the identity, functions, and relevance of these different types of amyloid proteins in fungal cells, and if they have roles in fitness or virulence.

While the bulk of data in this study implicates amyloid protein aggregates, both as the principal target of optotracers as well as the organelle in *C. albicans*, allowing the differentiation of yeast and biofilm lifestyles, we do not rule out the possible contribution of cell wall carbohydrates. The fungal cell wall is a highly dynamic organelle implicated in both survival and pathogenicity. Contributing to approximately 40% of the cell’s volume, the polysaccharide-rich cell wall holds a variety of antigens that confers the versatility of both immune evasion and recognition during interactions with a host. It also plays a central role in morphogenesis, adherence and biofilm formation. As the spectral profiles of optotracer bound to purified chitin and β-glucans extracts were respectively distinct, changes in the relative proportion of each carbohydrate are likely to be reflected during spectrophotometric analysis. Indeed, Fourier transform infrared spectroscopy (FTIR) studies have revealed that a transition of *C. albicans* from yeast to hyphae is associated with a 10% increase in β-1,3- glucans and a 20% reduction in β-1,6-glucans. Furthermore, β-glucans in the hyphal form are also structurally distinct, exhibiting a cyclical structure where the β-1,3-linked polymer backbone is decorated with β-1,6- linked side chains (Lowman et al., 2003). As the molecular mechanisms underlying cell wall remodeling during the transition from the yeast to the hyphal form are still largely unknown, optotracing may be a useful tool in deciphering this biological phenomenon and pathophysiology studies of this microbe. Future work into correlating optotracer optical signals with cell wall composition and architecture may also allow the detection of virulent strains and the prediction of susceptibilities, thereby guiding treatment and preventing the resistance developing arising from ineffective prescriptions.

## Data availability statement

The original contributions presented in the study are included in the article/**Supplementary Material**. Further inquiries can be directed to the corresponding author.

## Author contributions

FC, EK and AR-D designed research. EK and SJ performed experiments. EK, FC, AR-D and UE analyzed data. FC and EK wrote the manuscript. AR-D and UE contributed intellectually to editing and finalization of the manuscript. All authors contributed to the article and approved the submitted version.

## Funding

This work was supported by AIMES – Center for the Advancement of Integrated Medical and Engineering Sciences (www.aimes.se) at Karolinska Institutet (1-249/2019) and KTH Royal Institute of Technology (VF-2019-0110), Erling-Persson Family Foundation (4-1784/2016), Swedish Foundation for Strategic Research (SB12-00347), and Getinge AB (4-1599/2018).

## Acknowledgments

We thank Professor K. Peter R. Nilsson for his invaluable expertise on disease associated amyloid aggregates and spectral imaging.

## Conflict of interest

FC and AR-D are co-inventors of patents relevant to this work. Intellectual properties are owned by Richter Life Science Development AB, founded by AR-D. FC and UE are shareholders of Furcifer AB. FC, UE and AR-D have an engagement in Ebba Biotech AB, which commercializes optotracers for uses as described in this article.

The remaining authors declare that the research was conducted in the absence of any commercial or financial relationships that could be construed as a potential conflict of interest.

## Publisher’s note

All claims expressed in this article are solely those of the authors and do not necessarily represent those of their affiliated organizations, or those of the publisher, the editors and the reviewers. Any product that may be evaluated in this article, or claim that may be made by its manufacturer, is not guaranteed or endorsed by the publisher.
