# Supplementary material for: Optotracing for live selective fluorescence-based detection of Candida albicans biofilms
Source: Front Cell Infect Microbiol. 2022 Sep 2;12:981454. doi: 10.3389/fcimb.2022.981454 (PMC9478205; doi:10.3389/fcimb.2022.981454)

Supplementary Information

Optotracing for live selective fluorescence-based detection of *Candida albicans* biofilms

Elina Kärkkäinen^1,2^, Saga G. Jakobsson^1,3^, Ulrica Edlund ^1,3^, Agneta Richter-Dahlfors ^1,2^, Ferdinand X. Choong^1,2*^

^1^ AIMES - Center for the Advancement of Integrated Medical and Engineering Sciences Karolinska Institutet and KTH Royal Institute of Technology, Stockholm, Sweden

^2^ Department of Neuroscience , Karolinska Institutet , Solnavägen 9, SE-171 77, Stockholm, Sweden

^3^ Fibre and Polymer Technology, KTH Royal Institute of Technology, Teknikringen 56, 100 44 Stockholm, Sweden

*** Correspondence:**
Ferdinand X. Choong
xiankeng.choong@ki.se

**Keywords: Biofilm_1_, Optotracing_2_, Candida_3_, Cell wall_4_, Amyloid_5_**

**Page index**

Supplementary Figure 1 *Immunofluorescence analysis of nuclei and extracellular carbohydrates in yeast cells and biofilm*............................................................................................................................................................3

Supplementary Figure 2 *Immunofluorescence analysis of cell wall components of yeast cells and biofilm*..................................................................................................................................................................4

Supplementary Figure 3 *Immunofluorescence analysis of intracellular amyloid organelles in yeast cells and biofilm*..................................................................................................................................................................5

Supplementary Figure 4 *Analysis of fluorescence signals emitted by cell wall carbohydrates with and without optotracer*.............................................................................................................................................................6

Supplementary Figure 5 *Optotracing of cell walls of yeast cells and biofilm using Ebba680...........................*.7

Supplementary Figure 6 *Optotracing of amyloid organelles in yeast cells and biofilm*.........................*............9*

Supplementary Figure 7 *Analysis of fluorescence signals emitted by yeast cells and biofilm with and without* optotracer...........................................................................................................................................................10

Supplementary Figure 8 *Spectal image analysis of amyloid bodies in yeast cells and biofilm samples*...............................................................................................................................................................11

**Supplementary figure 1**

***Immunofluorescence analysis of nuclei and extracellular carbohydrates in yeast cells and biofilms***

Confocal microscopy showing surface attached yeast cells from 72 h incubation in SDB, co-stained with **(A)** DAPI and **(B)** Concanavalin A. **(C)** Showing merged image of both channels. Similarly, **c**onfocal microscopy showing surface attached biofilm from 72 h incubation in RPMI, co-stained with **(D)** DAPI and **(E)** Concanavalin A. **(F)** Showing merged image of both channels. Representative images of 3 independent experiments are shown.


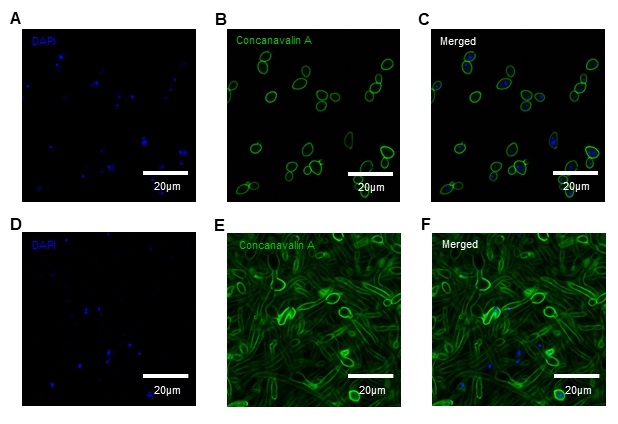


**Supplementary figure 2**

***Immunofluorescence analysis of cell wall components of yeast cells and biofilms***

Confocal microscopy showing surface attached yeast cells and from 72 h incubation in SDB, co-stained with **(A)** Calcofluor white and **(B)** Concanavalin A. **(C)** Showing merged image of both channels. Similarly, confocal microscopy showing surface attached biofilm from 72 h incubation in RPMI, co-stained with **(D)** Calcofluor white and **(E)** Concanavalin A. **(F)** Showing merged image of both channels. Representative images of 3 independent experiments are shown.


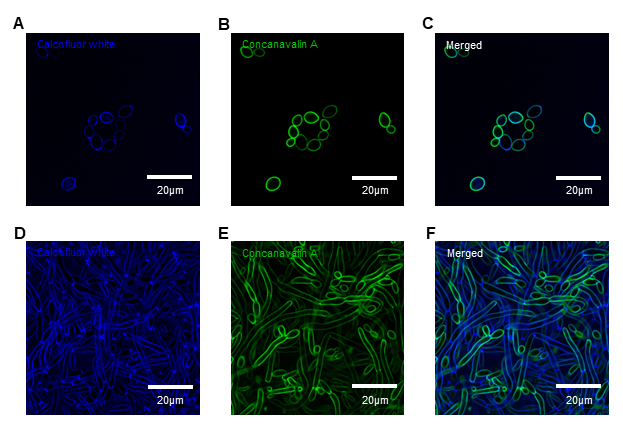


**Supplementary figure 3**

***Immunofluorescence analysis of intracellular amyloid organelles in yeast cells and biofilms***

Confocal microscopy showing surface attached yeast cells grown from 72h incubation in SDB, co-stained with **(A)** Calcofluor white and **(B)** Thioflavin S. **(C)** Showing merge images of both channels are shown. Similarly, confocal microscopy showing surface attached biofilm grown from 72h incubation in RPMI, co-stained with **(D)** Calcofluor white and **(E)** Thioflavin S. **(F)** Showing merged image of both channels. Representative images of 3 independent experiments are shown.


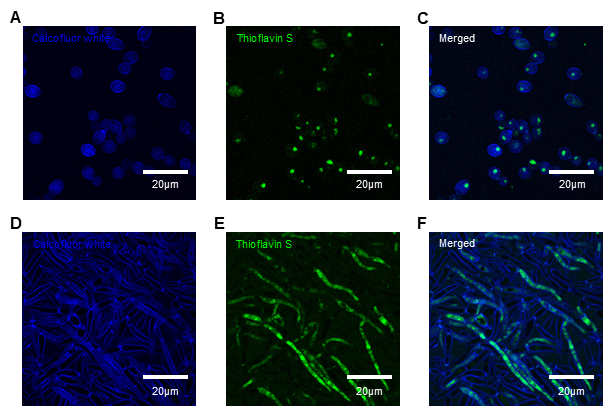


**Supplementary figure 4**

***Analysis of fluorescence signals emitted by cell wall carbohydrates with and without optotracer***

**(A)** N. Spec-plot of fluorescence collected from chitin (purple), mannan (blue), glucan (green) and Ebba680 (red). **(B)** N. Spec-plot of fluorescence collected from chitin + Ebba680 (purple), mannan + Ebba680 (blue), glucan + Ebba680 (green) and Ebba680 (red). **(C)** Fold change in fluorescence of each mixture with reference to the fluorescence of Ebba680 only.


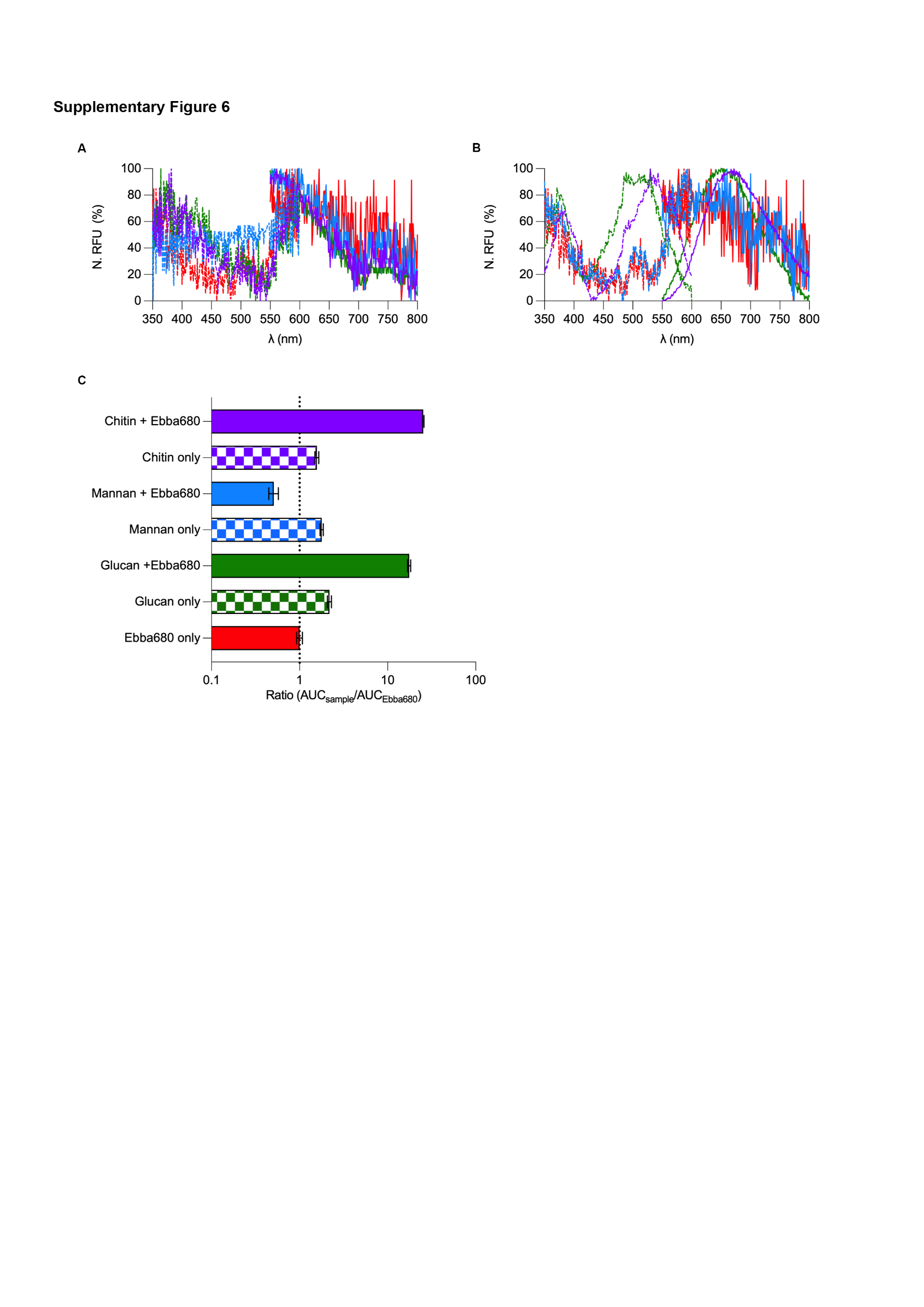


**Supplementary figure 5**

***Optotracing of cell walls of yeast cells and biofilms using Ebba680***

Confocal microscopy of surface attached yeast cells grown from 72 h incubation in Ebba680 supplemented SDB, co-stained with calcofluor white. Showing channels for **(A)** calcofluor white **(B)** Ebba680 and **(C)** the merged image of both channels. **(D)** Post-imaging enhancement of Ebba680 fluorescence to reveal tracer bound cell walls, **(E)** overlayed with Calcofluor white signal within the sample (bottom right). Similarly, confocal microscopy of surface attached biofilm grown from 72h incubation in Ebba680 supplemented RPMI, co-stained with calcofluor white, showing fluorescence from **(F)** Calcofluor white, **(G)** Ebba680 and **(H)** the merge of both channels. **(I)** Post-imaging enhancement of Ebba680 fluorescence to reveal tracer bound cell walls, **(J)** overlayed with Calcofluor white signal within the sample. Representative images of 3 independent experiments are shown.


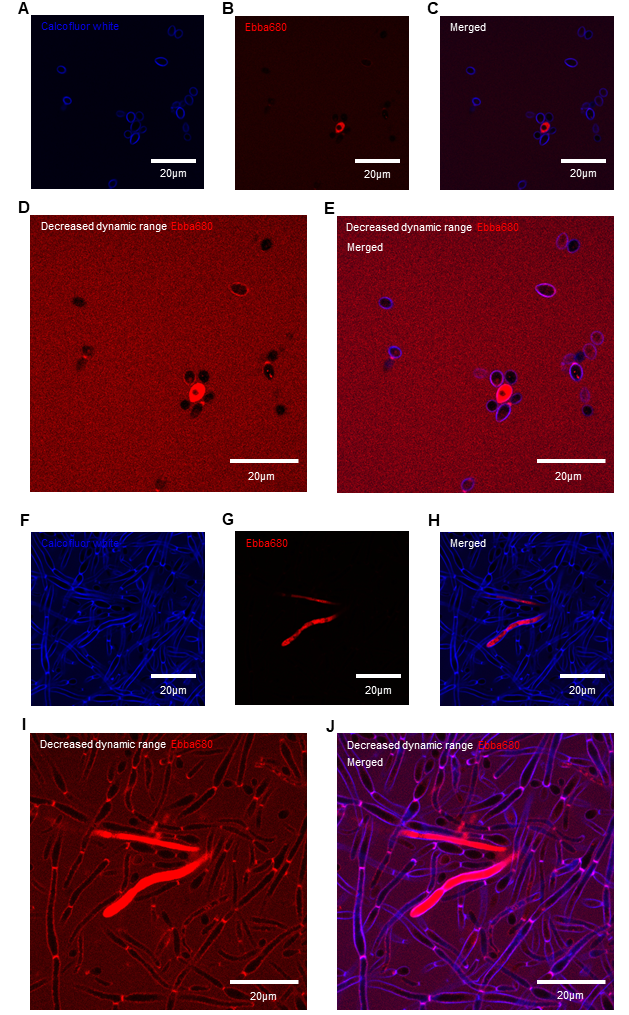


**Supplementary figure 6**

***Optotracing of amyloid organelles in yeast and biofilm culture***

Confocal microscopy of surface attached yeast cells grown from 72 h incubation in Ebba680 supplemented SDB, co-stained with calcofluor white and thioflavin S showing fluorescence from **(A)** Calcofluor white, **(B)** thioflavin S, **(C)** Ebba680 **(D)** and the merged image of all channels. Similarly, confocal microscopy of surface attached biofilm grown from 72 h incubation in Ebba680 supplemented RPMI, co-stained with calcofluor white and thioflavin S showing fluorescence from **(E)** Calcofluor white, **(F)** thioflavin S, **(G)** Ebba680 **(H)** and the merged image of all channels. Representative images of 3 independent experiments are shown.


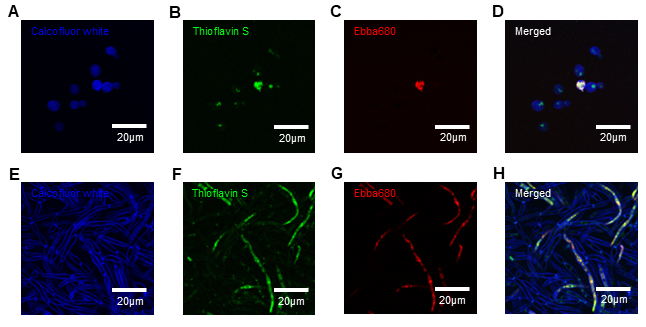


**Supplementary figure 7**

***Analysis of fluorescence signals emitted by yeast cells and biofilms with and without optotracers***

**(A)** Fold change in fluorescence of each mixture with reference to the fluorescence of SDB + Ebba680. **(B)** Fold change in fluorescence of each mixture with reference to the fluorescence of RPMI + Ebba680. All bars show mean of n=3, with 3 technical repeats. the standard deviation is shown.


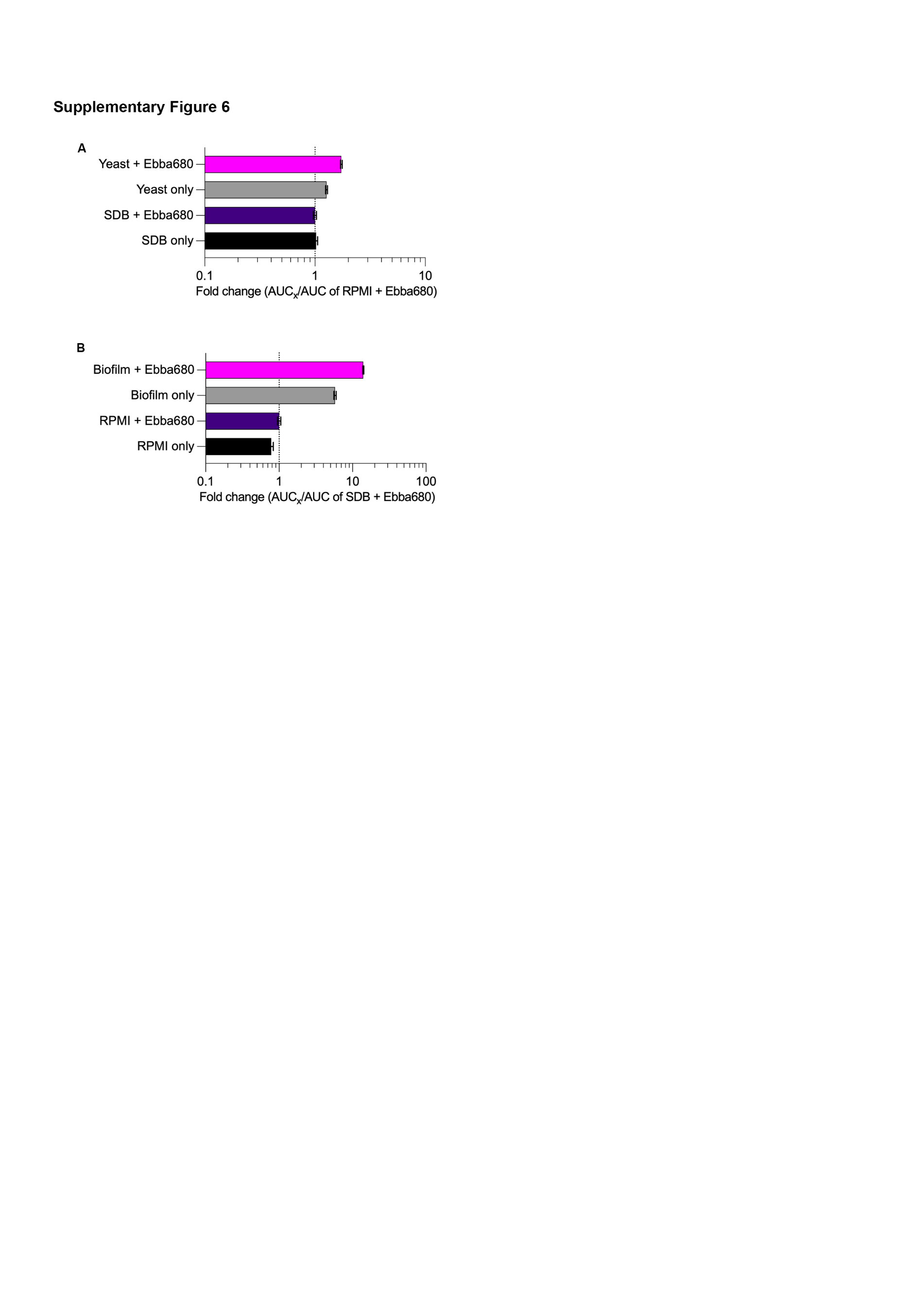


**Supplementary figure 8**

***Spectral image analysis of amyloid bodies in yeast and biofilm samples***

**(A)** Screenshot showing the regions of interest selected to visualize the emission spectra of bound Ebba680 are shown with a red crosshair from yeast and **(B)** biofilms. The analysis is representative of 2 independent experimental replicates.


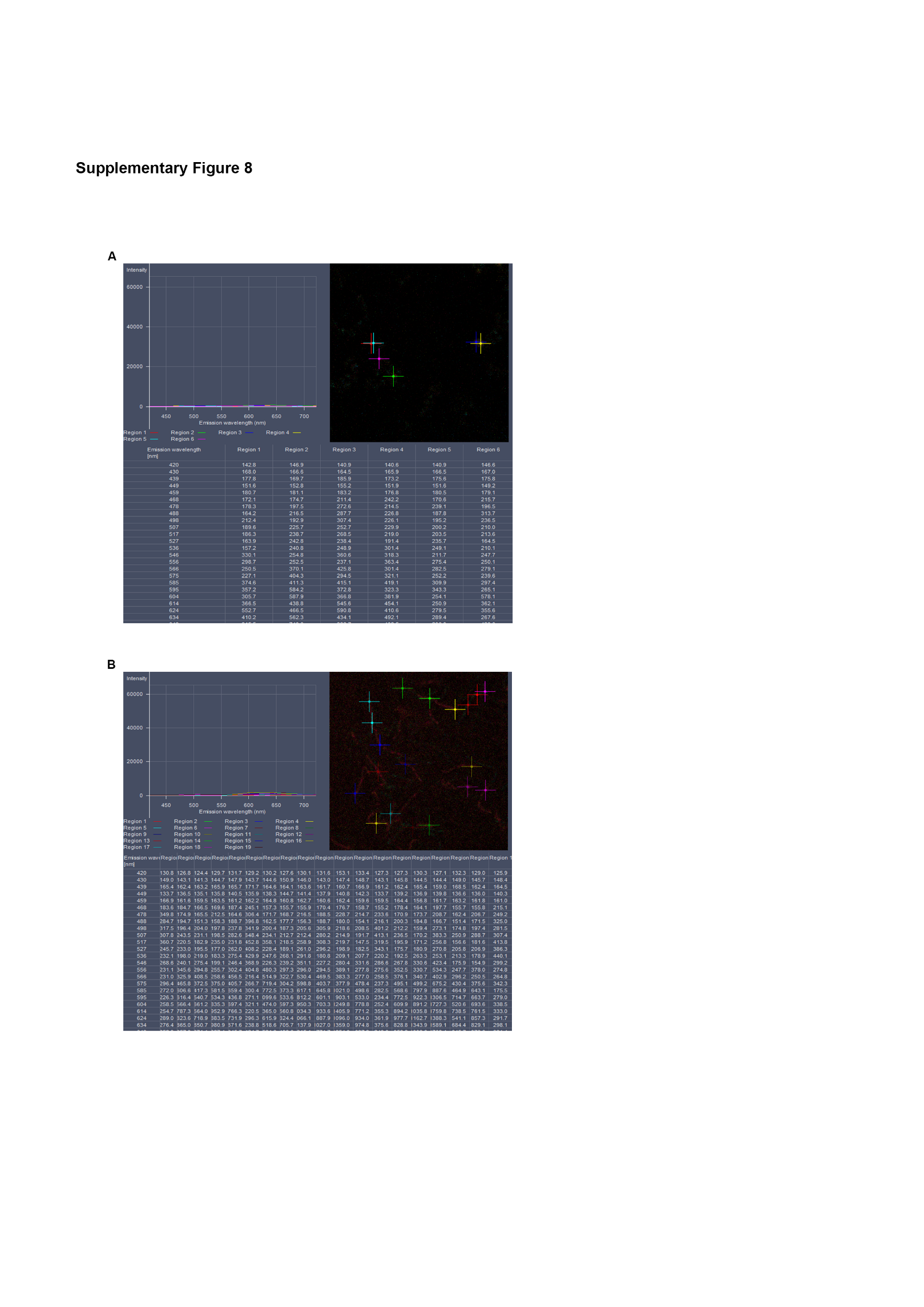

Supplement: Supplementary file 1 [file DataSheet_1.docx]
